# Quinone-Amino Acid Conjugates Targeting *Leishmania* Amino Acid Transporters

**DOI:** 10.1371/journal.pone.0107994

**Published:** 2014-09-25

**Authors:** Federica Prati, Adele Goldman-Pinkovich, Federica Lizzi, Federica Belluti, Roni Koren, Dan Zilberstein, Maria Laura Bolognesi

**Affiliations:** 1 Department of Pharmacy and Biotechnology, Alma Mater Studiorum University of Bologna, Bologna, Italy; 2 Faculty of Biology, Technion-Israel Institute of Technology, Haifa, Israel; National Center for Cell Science, India

## Abstract

The aim of the present study was to investigate the feasibility of targeting *Leishmania* transporters via appropriately designed chemical probes. *Leishmania donovani*, the parasite that causes visceral leishmaniasis, is auxotrophic for arginine and lysine and has specific transporters (LdAAP3 and LdAAP7) to import these nutrients. Probes **1–15** were originated by conjugating cytotoxic quinone fragments (**II** and **III**) with amino acids (i.e. arginine and lysine) by means of an amide linkage. The toxicity of the synthesized conjugates against *Leishmania* extracellular (promastigotes) and intracellular (amastigotes) forms was investigated, as well their inhibition of the relevant amino acid transporters. We observed that some conjugates indeed displayed toxicity against the parasites; in particular, **7** was identified as the most potent derivative (at concentrations of 1 µg/mL and 2.5 µg/mL residual cell viability was reduced to 15% and 48% in promastigotes and amastigotes, respectively). Notably, **6**, while retaining the cytotoxic activity of quinone **II**, displayed no toxicity against mammalian THP1 cells. Transport assays indicated that the novel conjugates inhibited transport activity of lysine, arginine and proline transporters. Furthermore, our analyses suggested that the toxic conjugates might be translocated by the transporters into the cells. The non-toxic probes that inhibited transport competed with the natural substrates for binding to the transporters without being translocated. Thus, it is likely that **6**, by exploiting amino acid transporters, can selectively deliver its toxic effects to *Leishmania* cells. This work provides the first evidence that amino acid transporters of the human pathogen *Leishmania* might be modulated by small molecules, and warrants their further investigation from drug discovery and chemical biology perspectives.

## Introduction

**Figure 1:**
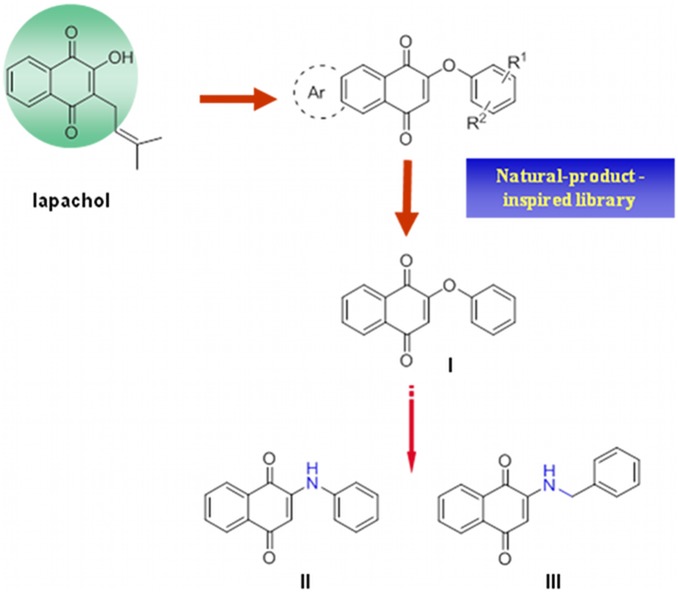
Design rationale to II and III. II and III were conceived to improve the chemical and metabolic stability of the natural-inspired compound **I**.

*Leishmania* is an obligate intracellular protozoan parasite responsible for leishmaniasis, a debilitating disease found in more than 88 countries, including all Mediterranean area [1]. Resulting infection in humans ranges from chronic skin ulcers, via erosive mucosal disease with progressive destruction of the nasopharynx and severe facial disfigurement, to a life-threatening systemic infection with hepatosplenomegaly [1]. Global yearly incidence of leishmaniasis approaches 2 million new cases with an estimated 59,000 deaths predominantly in India, Bangladesh, Nepal, and Sudan [2], representing a significant health problem in tropical and subtropical regions of the world. Despite the epidemiological importance in the developing world, such as the possible impact in Western societies, the drugs used for the treatment of this disease are unsatisfactory due to high cost, toxicity, problems in administration and resistance issues [3]. In view of the foregoing facts, there is still an urgent need for new and more effective drugs and innovative drug design strategies. In this context, biology oriented synthesis (BIOS) of natural product-derived and -inspired scaffolds may show promising results in finding new lead structures for chemical biology and medicinal chemistry research [4]. Indeed, this approach recognizes natural products as evolutionary selected and biologically pre-validated starting points in chemical space to be suitably deployed for library generation [4]. On this basis, the natural occurring lapachol was used to generate the natural product-inspired skeleton shown in Figure 1, and this was subjected to modification to yield a small library of potential hits [5]. In a phenotypic based screening, the most promising derivative resulted naphthoquinone **I** (Fig. 1), with an IC_50_ value (1.26 µM) against *Leishmania* axenic amastigotes only four time higher than the reference drug miltefosine (0.31 µM) [5]. However, it showed a selectivity index (SI) with respect to mammalian cells of only 4.7 [5]. This indicated **I** as a potential hit candidate for the development of compounds endowed with a better activity and, at the same time, better selectivity. Toward this goal, due to the lack of target information driving a target-based approach, we were forced toward a ligand-based design strategy. For this reason while fishing target(s) of **I** through chemical proteomics techniques [6], we envisaged a ‘targeting’ approach [7], [8], [9] as a suitable strategy to generate novel molecules endowed with an improved profile. Indeed, drug targeting, i.e. uptake of a drug via parasite-specific pathways, has been advocated as a chemotherapeutic strategy to selectively inhibit drug targets that have equally sensitive counterparts in the host [8].

It is well known that Trypanosomatid parasites (including *Trypanosoma* and *Leishmania* spp.) depend on their hosts for several nutrients, such as glucose, purines, amino acids, vitamins and lipids, necessary for their replication and survival [10]. In order to acquire these molecules, the parasites express plasma membrane transporters that mediate their intake [10]. These transporters can potentially be exploited to carry toxic compounds inside parasite cells, leading to selective toxicity delivered via selective transport systems [7], [8], [9]. Fairlamb [11] and co-workers first recognized the potential of the P2 purine transporter to carry drugs into trypanosomes [12]. From these findings, membrane transporters have been utilized by several medicinal chemists to design anti-parasitic agents with improved profiles [13], [14], [15], [16], [17], [18], [19], [20], [21], [22], [23], [24].

Furthermore, beside the selective accumulation of drugs into parasite cells, the design and development of new transporter-directed compounds could find application in the therapeutic area [25], [26]. In addition, from a chemical biology perspective, such compounds can be used as chemical probes to study the role of amino acid transporters as drug targets in *Leishmania*.

Transporter-directed compounds are typically chemical conjugates obtained by linking two moieties: (i) a so called *haptophore*, which is recognized by the transporter and (ii) a fragment that may have anti-parasitic activity (*toxophore*) [27].

Arginine (Arg) and lysine (Lys) are essential amino acids for *Leishmania*, whereas in mammals arginine is conditionally essential, depending on the health status and developmental stage of the organism [28]. Shaked-Mishan et al. and Inbar et al. have identified the arginine (LdAAP3) and lysine (LdAAP7) transporters from *Leishmania donovani*. These proteins translocate arginine and lysine at high-affinity and specificity. LdAAP3 expression and activity are highly dependent on arginine availability [29], [30], [31].

We hypothesized LdAAP3 and LdAAP7 can be targeted with small molecules by (i) selective delivery of cytotoxic molecules into the parasite, or (ii) by alteration of nutrients intake. In addition, this approach can be useful to elucidate the complex biology of parasite transporters.

**Figure 2:**
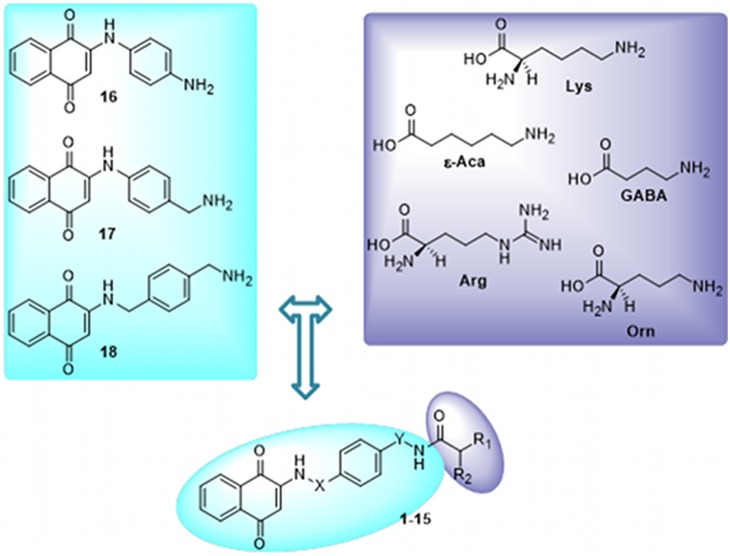
Design rationale to library conjugates 1–15. Compounds **1–15** were designed by linking **II** and **III** with several amino acid motifs.

Here, we designed new conjugates in which the leishmanicidal naphthoquinone moiety of **I** was coupled to Arg and Lys pendant groups (Figs. 1 and 2). We found, for the first time, that some conjugates inhibited transport and a few were cidal to *L. donovani* promastigotes and amastigotes via competitive inhibition with their natural substrates.

## Materials and Methods

### Chemistry

All reagents and solvents were purchased by Sigma Aldrich and used without further purification, unless otherwise stated. Melting points were measured on a Buchi SMP-20 apparatus and are uncorrected. Direct infusion ESI-MS spectra were recorded on Waters ZQ 4000 apparatus. NMR spectra were recorded on Varian VRX 200 and 400 MHz instruments. Chemical shifts (δ) are reported in parts per million (ppm) relative to tetramethylsilane (TMS), and spin multiplicities are given as s (singlet), br s (broad singlet), d (doublet), t (triplet), q (quartet), or m (multiplet). The elemental compositions of the compounds agreed to within ±0.4 % of the calculated value. When the elemental analysis is not included, crude compounds were used in the next step without further purification. Chromatographic separations were performed on silica gel (Kieselgel 40, 0.040–0.063 mm; Merck) by flash chromatography. Reactions were followed by thin-layer chromatography (TLC) on glass-backed pre-coated silica gel plates (0.25 mm, 60 F254; Merck), then visualized in an iodine chamber or with a UV lamp. The term “dried” refers to the use of anhydrous Na_2_SO_4_. Compounds were named following IUPAC rules as applied by ChemBioDraw Ultra 11.0.

### Synthesis of naphthoquinone 16–18

First, intermediates **19–21** were obtained by reacting 1,4-naphthoquinone (**22**) (0.67 g, 4.23 mmol) with *tert-*butyl 4-aminophenylcarbamate (**23**) (0.88 g, 4.23 mmol), *tert*-butyl 4-aminobenzylcarbamate (**24**) (0.94 g, 4.23 mmol), and *tert*-butyl 4-(aminomethyl)benzylcarbamate (**25**) (1 g, 4.23 mmol), respectively, in methanol (15 mL) at room temperature for 4 h. After precipitation, compounds **19** (0.90 g, 58%), **20** (0.87 g, 54%), and **21** (0.72 g, 48%) were collected by filtration and used in the next step without any further purification. Compounds **19** (0.67 g, 1.83 mmol), **20** (0.69 g, 1.83 mmol), and **21** (0.72 g, 1.83 mmol) were dissolved in dry CH_2_Cl_2_ (15 mL) and trifluoroacetic acid (TFA) (2.8 mL, 36.6 mmol) was added dropwise at 0°C. After stirring for 5 h at room temperature, the solvent was evaporated under vacuum, washing with heptane (3 times) to remove the excess of TFA. The crude extracts were then dissolved in water and washed with ether (3×20 mL). The water was made basic with Na_2_CO_3_ and extracted with CH_2_Cl_2_ (3×30 mL). The organic extracts were collected, dried and evaporated under vacuum to afford compounds **16** (0.44 g, 92%), **17** (0.48 g, 94%), and **18** (0.49 g, 92%). For characterization data of **19–21** and **16–18**, see File S1.

### Synthesis of activated amino acids 27–30

*N*_α_,*N*_ε_-Di-Boc-L-lysine hydroxysuccinimide ester (**26**) was commercially available. 4-(Boc-amino)butyric acid (**33**) and 6-(Boc-amino)caproic acid (**35**) were commercially available and were just subjected to the carboxylic group activation. L-Ornithine hydrochloride (**31**) (1 g, 5.93 mmol) was dissolved in NaOH (5 mL) and water (5 mL), then a solution of NaOH 4 N (2.73 mL) and a solution of (Boc)_2_O (2.98 g, 13.64 mmol) in 5 mL of THF were added dropwise and stirred for 6 h at room temperature. THF was evaporated and the aqueous solution was made acidic with KHSO_4_ and extracted with ethyl acetate (2×20 mL). The organic extracts were collected, dried over Na_2_SO_4_ and evaporated under vacuum. The crude was crystallized by ethyl acetate/cyclohexane to give **34** (1.67 g, 85%). *N*_α_-Boc-L-arginine (**32**) (1 g, 3.65 mmol) was dissolved in NaOH 2N (17.5 mL) and dioxane (15 mL) at 0°C. Di-*tert*-butyl dicarbonate (1.64 g, 7.51 mmol) was added and the resulting suspension was stirred at room temperature for 3 h. Then, further di-*tert*-butyl dicarbonate (0.82 g, 3.72 mmol) in dioxane (7.5 mL) was added, stirring for additional 39 h. The pH was adjusted to 7 with KHSO_4_ and the reaction mixture extracted with ethyl acetate (3×20 mL). The organic extracts were collected, dried over Na_2_SO_4_ and evaporated under vacuum. The crude was purified by flash chromatography eluting with CHCl_3_/MeOH (9∶1) to afford **36** (1.32 g, 76%).

To a solution of **33** (0.5 g, 2.46 mmol), **34** (0.82 g, 2.46 mmol), **35** (0.57 g, 2.46 mmol), and **36** (1.17 g, 2.46 mmol) in CH_2_Cl_2_ (15 mL) *N*-hydroxysuccinimide (NHS) (0.28 g, 2.46 mmol) was added. When necessary, a small amount of THF (ca. 4 mL) was added to improve solubility. *N*,*N*'-Dicyclohexylcarbodiimide (DCC) (0.51 g, 2.46 mmol) was added to the mixture, followed by a catalytic amount of 4-dimethylaminopyridine (DMAP) (4 mg). The reactions were stirred under nitrogen at room temperature overnight. The formed white solid was removed by filtration and the filtrates were evaporated to dryness. The resulting residue was taken up with ethyl acetate (30 mL) and extracted with 10% NaHCO_3_ (3×20 mL). The organic layers were dried over Na_2_SO_4_ and concentrated *in vacuo* to afford the protected and activated amino acids **27** (0.63 g, 85%), **28** (0.64 g, 60%), **29** (0.74 g, 92%) and **30** (0.62 g, 44%). For characterization data of **34–36** and **27–30**, see File S1.

### Synthesis of amino acid-quinone conjugates 1–15

The final coupling was carried out using a Carousel 12 Plus reaction station. The protected and activated amino acids **26** (0.067 g, 0.15 mmol), **27** (0.045 g, 0.15 mmol), **28** (0.064 g, 0.15 mmol), **29** (0.049 g, 0.15 mmol) and **30** (0.085 g, 0.15 mmol) were dissolved in dry CH_2_Cl_2_ (4 mL). Compounds **17** (0.042 g, 0.15 mmol) and **18** (0.044 g, 0.15 mmol) were then added and the resulting solutions were stirred for 5 h at room temperature. Then, removal of *N*-Boc protecting groups was carried out by dropwise addition of TFA at 0°C (10 eq each Boc protecting group). The resulting solution was left to stir at room temperature for 3 h. The solvent was then evaporated under vacuum washing with heptane (3 times) to remove the excess of TFA. The solid residues were repeatedly triturated with ether, filtered and crystallized from ethanol/ether to afford the final compounds **6** (0.063 g, 66%), **7** (0.046 g, 64%), **8** (0.056 g, 60%), **9** (0.041 g, 54%), **10** (0.076 g, 63%), **11** (0.056 g, 58%), **12** (0.042 g, 57%), **13** (0.053 g, 56%), **14** (0.042 g, 54%), **15** (0.082 g, 68%) as trifluoroacetate salts.

A modified procedure was followed for the synthesis of **1–5**. **16** (0.04 g, 0.15 mmol) was dissolved in dioxane (0.5 mL), then DMAP (0.018 g, 0.15 mmol) and **26** (0.067 g, 0.15 mmol), **27** (0.045 g, 0.15 mmol), **28** (0.064 g, 0.15 mmol), **29** (0.049 g, 0.15 mmol) and **30** (0.085 g, 0.15 mmol) were added. The solutions were stirred at 80°C for 24 h, and, after cooling to room temperature, the solvent was evaporated under vacuum. The removal of *N*-Boc protecting group was carried out as described for **6–15**, affording **1** (0.052 g, 55%), **2** (0.033 g, 48%), **3** (0.045 g, 49%), **4** (0.032 g, 43%), **5** (0.056 g, 51%) as trifluoroacetate salts. For elemental analyses of compounds **1–15** see File S1.

### 2,6-diamino-N-(4-(1,4-dioxo-1,4-dihydronaphthalen-2-ylamino)phenyl)hexanamide trifluoroacetate salt (**1**)

Red solid; 55% yield; mp: 135°C; ^1^Η NMR (CD_3_OD, 400 MHz): δ 1.56–1.59 (m, 2H), 1.73–1.78 (m, 2H), 1.98–2.06 (m complex, 2H), 2.97 (t, *J* = 7.6, 2H), 4.07 (t, *J* = 6.4, 1H), 6.17 (s, 1H), 7.37 (d, *J* = 8.8, 2H), 7.74–7.81 (m, 3H), 7.82 (t, J = 6.8, 1H), 8.04 (d, *J* = 6.8, 1H), 8.14 (d, *J* = 6.8, 1H); ^13^C-NMR (CD_3_OD, 100 MHz): δ 20.2, 25.5, 29.5, 37.5, 52.2, 99.9, 110.1, 119.4, 122.8, 124.1, 124.8, 129.4, 131.1, 133.2, 165.6; MS (ESI^+^) *m/z*: 393 [M + H]^+^.

### 4-amino-N-(4-(1,4-dioxo-1,4-dihydronaphthalen-2-ylamino)phenyl)butanamide trifluoroacetate salt (**2**)

Red solid; 48% yield; mp: 190°C; ^1^H NMR (CD_3_OD, 200 MHz): δ 1.98–2.07 (m, 2H), 2.59 (t, *J* = 6.6, 2H), 3.06 (t, *J* = 6.8, 2H), 6.16 (s, 1H), 7.34 (d, *J* = 7.6, 2H), 7.69 (d, *J* = 7.6, 2H), 7.73–7.86 (m, 2H), 8.04 (d, *J* = 7.4, 1H), 8.14 (d, *J* = 7.4, 1H); ^13^C-NMR (CD_3_OD, 50 MHz): δ 23.3, 32.7, 39.2, 102.0, 124.1, 125.2, 126.7, 128.2, 131.0, 132.3, 133.2, 134.9, 136.8, 182.4; MS (ESI^+^) *m/z*: 350 [M + H]^+^.

### 2,5-diamino-N-(4-(1,4-dioxo-1,4-dihydronaphthalen-2-ylamino)phenyl)pentanamide trifluoroacetate salt (**3**)

Red solid; 49% yield; mp: 185°C; ^1^H NMR (CD_3_OD, 400 MHz): δ 1.83–1.86 (m, 2H), 2.01–2.07 (m, 2H), 3.01 (t, *J* = 8.0, 2H), 4.09 (t, *J* = 8.0, 1H), 6.14 (s, 1H), 7.36 (d, *J* = 8.8, 2H), 7.74 (d, *J* = 8.8, 2H), 7.78–7.82 (m, 2H), 8.02 (d, *J* = 7.6, 1H), 8.11 (d, *J* = 7.6, 1H); ^13^C-NMR (CD_3_OD, 100 MHz): δ 22.9, 28.5, 38.9, 53.3, 101.5, 120.9, 124.3, 125.6, 126.3, 132.6, 134.6, 135.6, 166.8, 184.6; MS (ESI^+^) *m/z*: 379 [M + H]^+^.

### 6-amino-N-(4-(1,4-dioxo-1,4-dihydronaphthalen-2-ylamino)phenyl)hexanamide trifluoroacetate salt (**4**)

Red solid; 43% yield; mp: 173°C; ^1^H NMR (CD_3_OD, 200 MHz): δ 1.48–1.53 (m, 2H), 1.69–1.78 (m, 4H), 2.46 (t, *J* = 6.8, 2H), 2.96 (t, *J* = 7.4, 2H), 6.17 (s, 1H), 7.34 (d, *J* = 8.4, 2H), 7.69 (d, *J* = 8.4, 2H), 7.73–7.84 (m, 2H), 8.05 (d, *J* = 7.0, 1H), 8.15 (d, *J* = 7.0, 1H); ^13^C-NMR (CD_3_OD, 50 MHz): δ 23.2, 24.2, 25.8, 34.8, 37.8, 41.1, 44.3, 99.2, 124.7, 124.9, 126.2, 126.8, 130.3, 133.3, 136.8; MS (ESI^+^) *m/z*: 378 [M + H]^+^.

### 2-amino-N-(4-(1,4-dioxo-1,4-dihydronaphthalen-2-ylamino)phenyl)-5-guanidinopentanamide trifluoroacetate salt (**5**)

Red solid; 51% yield; mp: 181°C; ^1^H NMR (CD_3_OD, 400 MHz): δ 1.76–1.79 (m, 2H), 2.01–2.07 (m, 2H), 3.28 (t, *J* = 7.2, 2H), 4.09 (t, *J* = 6.4, 1H), 6.17 (s, 1H), 7.38 (d, *J* = 8.8, 2H), 7.74–7.78 (m, 3H), 7.83 (t, *J* = 6.4, 1H), 8.05 (d, *J* = 6.4, 1H), 8.14 (d, *J* = 6.4, 1H); ^13^C-NMR (CD_3_OD, 100 MHz): δ 22.7, 27.1, 39.1, 52.0, 99.9, 119.4, 122.8, 124.1, 124.8, 131.1, 131.8, 133.2, 134.1, 165.4; MS (ESI^+^) *m/z*: 421 [M + H]^+^.

### 2,6-diamino-N-(4-(1,4-dioxo-1,4-dihydronaphthalen-2-ylamino)benzyl)hexanamide trifluoroacetate salt (**6**)

Red solid; 66% yield; mp: 123°C; ^1^H NMR (CD_3_OD, 200 MHz): δ 1.45–1.50 (m, 2H), 1.70–1.73 (m, 2H), 1.88–1.91 (m, 2H), 2.93 (t, *J* = 7.2, 2H), 3.92 (t, *J* = 6.4, 1H), 4.39 (s, 2H), 6.18 (s, 1H), 7.34–7.48 (m, 4H), 7.72–7.86 (m, 2H), 8.05 (d, *J* = 7.0, 1H), 8.14 (d, *J* = 7.0, 1H); ^13^C-NMR (CD_3_OD, 50 MHz): δ 20.1, 25.8, 29.9, 37.5, 52.2, 99.8, 110.6, 119.0, 123.1, 124.7, 125.2, 129.8, 131.7, 133.3, 165.2; MS (ESI^+^) *m/z*: 407 [M + H]^+^.

### 4-amino-N-(4-(1,4-dioxo-1,4-dihydronaphthalen-2-ylamino)benzyl)butanamide trifluoroacetate salt (**7**)

Red solid; 64% yield; mp: 158–160°C; ^1^H NMR (CD_3_OD, 400 MHz): δ 1.91–1.98 (m, 2H), 2.42 (t, *J* = 7.2, 2H), 2.98 (t, *J* = 7.2, 2H), 4.39 (s, 2H), 6.17 (s, 1H), 7.32 (d, *J* = 8.8, 2H), 7.38 (d, *J* = 8.8, 2H), 7.71–7.82 (m, 2H), 8.02 (d, *J* = 7.6, 1H), 8.12 (d, *J* = 7.6, 1H); ^13^C-NMR (CD_3_OD, 100 MHz): δ 23.2, 32.5, 39.2, 42.5, 101.6, 123.7, 125.6, 126.3, 128.7, 130.9, 132.6, 133.3, 134.7, 136.6, 182.6; MS (ESI^+^) *m/z*: 364 [M + H]^+^.

### 2,5-diamino-N-(4-(1,4-dioxo-1,4-dihydronaphthalen-2-ylamino)benzyl)pentanamide trifluoroacetate salt (**8**)

Red solid; 60% yield; mp: 190–192°C; ^1^H NMR (CD_3_OD, 200 MHz): δ 1.78–1.98 (m, 4H), 3.01 (t, *J* = 7.6, 2H), 3.94 (t, *J* = 6.4, 1H), 4.49 (s, 2H), 6.20 (s, 1H), 7.37–7.48 (m, 4H), 7.75–7.84 (m, 2H), 8.05 (d, *J* = 7.0, 1H), 8.15 (d, *J* = 7.0, 1H); ^13^C-NMR (CD_3_OD, 50 MHz): δ 23.1, 28.2, 40.0, 53.8, 101.2, 121.0, 124.3, 125.2, 126.5, 132.2, 134.3, 135.1, 166.8, 184.8; MS (ESI^+^) *m/z*: 393 [M + H]^+^.

### 6-amino-N-(3-(1,4-dioxo-1,4-dihydronaphthalen-2-ylamino)benzyl)hexanamide trifluoroacetate salt (**9**)

Red solid; 54% yield; mp: 138–140°C; ^1^H NMR (CD_3_OD, 200 MHz): δ 1.42–1.46 (m, 2H), 1.68–1.71 (m, 4H), 2.32 (t, *J* = 6.8, 2H), 2.94 (t, *J* = 7.4, 2H), 4.41 (s, 2H), 6.19 (s, 1H), 7.35–7.40 (m, 4H), 7.75–7.84 (m, 2H), 8.05 (d, *J* = 7.2, 1H), 8.15 (d, *J* = 7.2, 1H); ^13^C-NMR (CD_3_OD, 50 MHz): δ 24.0, 24.7, 25.9, 33.2, 37.9, 41.9, 44.2, 98.2, 124.9, 125.1, 125.9, 126.8, 130.9, 133.2, 136.8; MS (ESI^+^) *m/z*: 392 [M + H]^+^.

### 2-amino-N-(4-(1,4-dioxo-1,4-dihydronaphthalen-2-ylamino)benzyl)-5-guanidinopentanamide trifluoroacetate salt (**10**)

Red solid; 63% yield; mp: 178°C; ^1^H NMR (CD_3_OD, 400 MHz): δ 1.63–1.70 (m, 2H), 1.89–1.97 (m, 2H), 3.22 (t, *J* = 6.8, 2H), 3.92 (t, *J* = 6.4, 1H), 4.44 (s, 2H), 6.16 (s, 1H), 7.33 (d, *J* = 8.4, 2H), 7.41 (d, *J* = 8.4, 2H), 7.72 (t, *J* = 7.6, 1H), 7.79 (t, *J* = 7.6, 1H), 8.01 (d, *J* = 7.6, 1H), 8.10 (d, *J* = 7.6, 1H); ^13^C-NMR (CD_3_OD, 100 MHz): δ 22.7, 27.1, 38.9, 41.1, 51.4, 100.0, 122.2, 124.1, 124.8, 127.4, 129.3, 131.1, 133.2, 134.4, 135.8, 167.1; MS (ESI^+^) *m/z*: 435 [M + H]^+^.

### 2,6-diamino-N-(4-((1,4-dioxo-1,4-dihydronaphthalen-2-ylamino)methyl)benzyl)hexanamide trifluoroacetate salt (**11**)

Orange solid; 58% yield; mp:142–145°C; ^1^H NMR (CD_3_OD, 200 MHz): δ 1.27–1.62 (m complex, 6H), 2.70 (t, *J* = 6.2, 2H), 3.42 (t, *J* = 6.4, 1H), 4.39 (s, 2H), 4.45 (s, 2H), 5.77 (s, 1H), 7.30 (s, 4H), 7.60–7.78 (m, 2H), 8.05–8.12 (m, 2H); ^13^C-NMR (CD_3_OD, 50 MHz): δ 20.2, 26.2, 30.1, 37.9, 52.9, 99.9, 110.2, 118.9, 123.5, 124.4, 125.2, 130.0, 131.1, 133.4, 165.9; MS (ESI^+^) *m/z* (M+H)^+^; MS (ESI^+^) *m/z*: 421 [M + H]^+^.

### 4-amino-N-(4-((1,4-dioxo-1,4-dihydronaphthalen-2-ylamino)methyl)benzyl)butanamide trifluoroacetate salt (**12**)

Orange solid; 57% yield; ^1^H NMR (CD_3_OD_,_ 200 MHz): δ 1.88–1.97 (m, 2H), 2.39 (t, *J* = 7, 2H), 2.96 (t, *J* = 6.6, 2H), 4.37 (s, 2H), 4.49 (s, 2H), 5.60 (s, 1H), 7.32 (s, 4H), 7.69–7.89 (m, 2H), 7.96–8.11 (m, 2H); ^13^C-NMR (CD_3_OD, 50 MHz): δ 23.7, 32.5, 39.8, 43.1, 101.8, 123.9, 125.9, 126.8, 128.2, 130.2, 132.8, 133.8, 134.3, 136.3, 182.4; MS (ESI^+^) *m/z*: 378 [M + H]^+^.

### 2,5-diamino-N-(4-((1,4-dioxo-1,4-dihydronaphthalen-2-ylamino)methyl)benzyl)pentanamide trifluoroacetate salt (**13**)

Orange solid; 56% yield, mp: 183–185°C; ^1^H NMR (CD_3_OD, 400 MHz): δ 1.71–1.77 (m, 2H), 1.87–1.93 (m, 2H), 2.95 (t, *J* = 7.2, 2H), 3.87 (t, *J* = 6.8, 1H), 4.41 (s, 2H), 4.48 (s, 2H), 5.57 (s, 1H), 7.33 (s, 4H), 7.69 (t, *J* = 7.6, 1H), 7.76 (t, *J* = 7.6, 1H), 7.97 (d, *J* = 7.6, 1H), 8.06 (d, *J* = 7.6, 1H); ^13^C-NMR (CD_3_OD, 100 MHz): δ 22.9, 28.4, 38.8, 42.9, 45.5, 52.7, 100.4, 125.6, 126.1, 127.4, 128.2, 132.3, 134.7; MS (ESI^+^) *m/z*: 407 [M + H]^+^.

### 6-amino-N-(4-((1,4-dioxo-1,4-dihydronaphthalen-2-ylamino)methyl)benzyl)hexanamide trifluoroacetate salt (**14**)

Orange solid; 54% yield; mp: 179°C; ^1^H NMR (CD_3_OD, 400 MHz): δ 1.38–1.42 (m, 2H), 1.64–1.69 (m, 4H), 2.27 (t, *J* = 7.6, 2H), 2.92 (t, *J* = 7.2, 2H), 4.36 (s, 2H), 4.49 (s, 2H), 5.62 (s, 1H), 7.29–7.35 (m, 4H), 7.65 (t, *J* = 6.4, 1H), 7.72 (t, *J* = 6.4, 1H), 8.13 (d, *J* = 6.4, 1H), 8.18 (d, *J* = 6.4, 1H); ^13^C-NMR (CD_3_OD, 100 MHz): δ 23.5, 24.2, 25.5, 33.8, 37.8, 41.1, 44.0, 98.8, 124.1, 124.6, 125.7, 126.4, 130.8, 133.1, 136.8; MS (ESI^+^) *m/z*: 406 [M + H]^+^.

### 2-amino-N-(4-((1,4-dioxo-1,4-dihydronaphthalen-2-ylamino)methyl)benzyl)-5-guanidinopentanamide trifluoroacetate salt (**15**)

Orange solid; 68% yield; mp: 178°C; ^1^H NMR (CD_3_OD, 400 MHz): δ 1.61–1.65 (m, 2H), 1.67–1.92 (m, 2H), 3.18 (t, *J* = 7.2, 2H), 3.89 (t, *J* = 8.8, 1H), 4.38 (s, 2H), 4.44 (s, 2H), 5.55 (s, 1H), 7.31 (s, 4H), 7.63 (t, *J* = 7.2, 1H), 7.72 (t, *J* = 7.2, 1H), 7.92 (d, *J* = 7.2, 1H), 8.01 (d, *J* = 7.2, 1H); ^13^C-NMR (CD_3_OD, 100 MHz): δ 24.2, 28.6, 40.5, 42.9, 45.5, 47.2, 100.4, 125.6, 126.1, 127.4, 128.2, 130.8, 132.3, 133.5, 134.7, 136.4, 137.6, 149.4, 157.5, 168.6, 181.4, 183.6; MS (ESI^+^) *m/z*: 449 [M + H]^+^.

### Biological assays of **I,** **II** and **III**

*In vitro* activity against axenic amastigotes of *L. donovani* (strain MHOM/ET/67/L82), and cytotoxicity assessment against L6 cells were determined as previously reported [32].

### *Leishmania* cell cultures

*L. donovani* MHOM/S.D./00/1 S; 1SR cell line was used [33]. Promastigotes and amastigotes were grown in axenic cultures and maintained as described previously [33], [34].

### Thymidine incorporation assays

Assays were carried out as described in Shaked-Mishan et al.[35]. Briefly, to 1×10^7^ cells 1 Ci/mL of [^3^H]-thymidine was added in 24-well ELISA plates. After a 3 h incubation for promastigotes and 24h for amastigotes, the cells were transferred to a 2 mL tube and washed in 2 mL cold PBS. Then they were suspended in 2 mL lysis buffer (140 mM NaCl, 1.5 mM MgCl_2_, 10 mM Tris–HCl pH 8.6 and 0.5% NP-40). The suspension was vortexed for 10 seconds and then centrifuged for three minutes (6000 g at 4°C). The nuclei-containing pellet was suspended in 2 mL ice-cold 10% trichloroacetic acid (TCA), kept at 4°C for at least 20 minutes and subsequently filtered through GF/C filters (Whatman International Ltd.). The filters were washed with 5 mL 10% TCA and then with 5 mL 95% ethanol. The filters were then subjected to scintillation counting.

### Transport assays

Uptake of 25 µM [^3^H]-arginine (600 mCi/mmol), 10 µM [^3^H]-lysine (1.0 Ci/mmol), or 1 mM [^3^H]-proline (8.0 mCi/mmol) into mid log phase parasites was determined using the rapid filtration technique [36]. To determine initial rates of transport, transport measurements were performed on 1×10^8^ promastigotes or amastigotes, which were exposed to radiolabel for up to 5 minutes. The amount of radiolabel associated with the cells was linear with time over the 5 minutes time course of the transport assay. Quinone-amino acid conjugates dissolved in DMSO were added to the cells and pre-incubated for 10 minutes prior to the beginning of transport measurement. Pre-incubation was carried out at 30°C for promastigotes and 37°C for amastigotes.

## Results

### Conjugates design rationale

Quinones **II** and **III** (Fig. 1) were selected as the *toxophores* to be conjugated with the proper *haptophoric* amino acids. Thus, **II** and **III** were tested against *Leishmania* axenic amastigotes (strain MHOM-ET-67/L82). Despite they showed leishmanicidal activities (6.02 µM and 8.74 µM, respectively) lower than **I** (1.26 µM), while retaining the same toxicity against mammalian L6 cells (3.73 µM, 7.03 µM, 5.92 µM respectively), **II** and **III** were preferred for the further conjugation approach. This is because the replacement of the ether linkage of **I** with the amino isosteric groups of **II** and **III** might have a positive effect on the chemical and metabolic stability of phenoxy compound.

**Figure 3:**
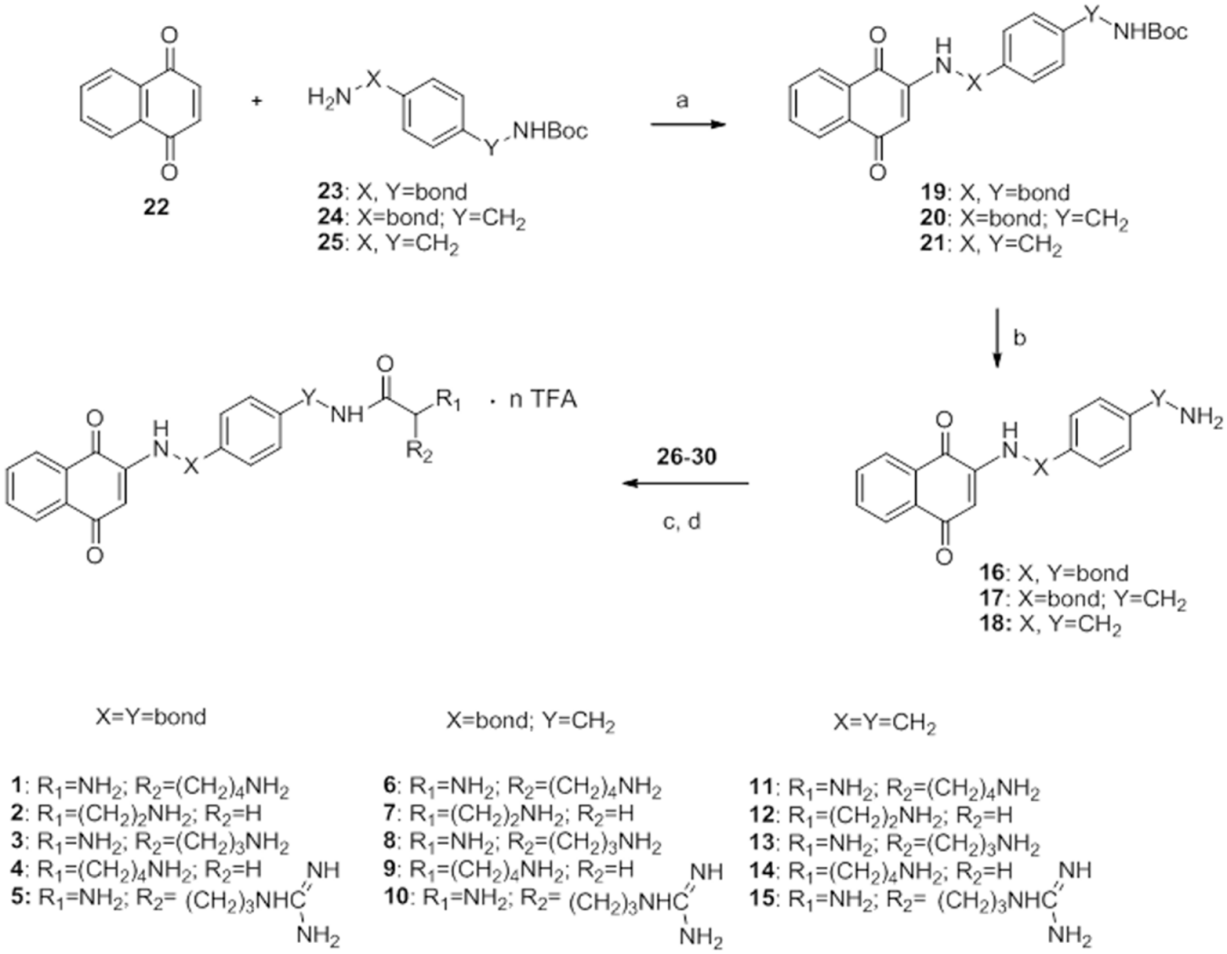
Synthesis of quinone-amino acid conjugates 1–15. Reagents and Conditions: a) MeOH, rt, 4 h; b) TFA, DCM, rt, 5 h; c) dioxane, DMAP, reflux, 24 h for **1–5**; CH_2_Cl_2_, rt 5 h for **6–15**; d) TFA, CH_2_Cl_2,_ rt.

Accordingly, the conjugates **1–15**, targeting three *Leishmania donovani* amino acid transporters (i.e. LdAAP3 LdAAP7 and proline transporter LdAAP24), were designed by linking **II** and **III** with several amino acid motifs (see Fig. 2 for design strategy). To this aim, we generated three quinones (**16–18**) bearing a free amino group in position 4 of the benzene ring that could be coupled with the carboxyl group of the amino acid through amide formation. By conjugating **16–18** with five amino acid **haptophores,** a combinatorial library of fifteen derivatives was generated (**1–15**). In addition to the Lys (**1**, **6**, **11**; see Fig. 3 for structures) and Arg (**5**, **10**, **15**) motifs, we selected the dibasic α-amino acid ornithine (Orn) (**3**, **8**, **13**), and the two ω-amino acids 4-amino-butiric (GABA) (**2**, **7**, **12**) and 6-amino-caproic (ε-Aca) (**4**, **9**, **14**) acids. This was to create molecular diversity and to obtain preliminary Structure Activity Relationship (SAR) within the series.

### Conjugates synthesis

**II** [37] and **III** [36], [38] were prepared according to reported procedures. The novel synthetic route developed for the preparation of quinone-amino acid conjugates **1–15** is depicted in Figure 3. After proper optimization conditions, all the reactions steps were carried out in a parallel fashion using a carousel workstation, with significant improvement of the overall efficiency. The first step was the synthesis of quinones **16–18** (Fig. 3). The Boc amino-protecting group was selected along all the procedure, because of its compatibility with the stability of the amino-quinone bond. Michael-type addition of amines onto *p*-quinones was adopted as the *C*–*N* coupling reaction. There are several reports on the use of this reaction to access 1,4-naphthoquinones possessing a substituted amino group in the 2-position [39], [40], [41]. However, especially for the transformation of less reactive anilines, catalysts [42], high temperature [37], and long reaction time [36], [38] are often required. We developed a simple and straightforward experimental protocol which led to **19–21** in good yields and in parallel fashion. In details, they were synthesized by reacting 1,4-naphthoquinone (**22**) with three monoBoc-diamines, namely *tert*-butyl 4-aminophenylcarbamate (**23**), *tert*-butyl 4-aminobenzylcarbamate (**24**) and *tert*-butyl 4-(aminomethyl)benzylcarbamate (**25**) in a 1∶1 ratio in MeOH for 4 h. Removal of *N*-Boc protecting groups by TFA afforded compounds **16–18** quantitatively. Then, **1–15** were obtained in a one-pot sequence that involves the coupling of **16–18** to the five protected amino acids (**26–30**), activated as NHS esters, and the subsequent removal of the *N*-Boc protecting groups by acidic treatment. NHS esters are widely used as coupling agents in peptide synthesis and were found suitable for our amide formation [43]. However, the lower reactivity of the aniline group of **16** with respect to the benzyl amine of **17** and **18,** required more drastic coupling conditions [44]. Reaction between **16** and amino acids **26–30** was carried out in dioxane at 80°C for 24 h using DMAP as catalyst, while for **17–18** the reaction was easily carried out in dry CH_2_Cl_2_ for 5 h at room temperature.

**Figure 4:**
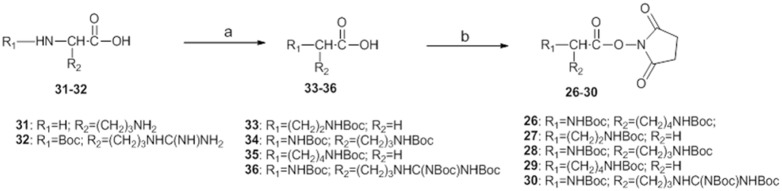
Synthesis of activated amino acids 26–30. Reagents and Conditions: a) (Boc)_2_O, NaOH, THF, rt for **31**; (Boc)_2_O, NaOH, dioxane, 0°C for **32**; b) DCC, DMAP, CH_2_Cl_2_, THF.

NHS esters were prepared accordingly to standard peptide synthesis procedures; the available amino acids were first protected on the amino groups and then activated on the carboxylic group (Fig. 4). The amino groups of L-ornithine (**31**) and *N*_α_-Boc-L-arginine (**32**) were protected by using slightly different conditions: for **31** the reaction was carried out in THF using (Boc)_2_O and NaOH, while for **32** in dioxane [45] at 0°C. 4-(Boc-amino)butyric acid (**33**) and 6-(Boc-amino)caproic acid (**35**) were already commercially available. Then, the carboxylic group of **33–36** was activated by using NHS in presence of DCC and DMAP to afford derivatives **27–30**. *N*_α_,*N*_ε_-Di-Boc-L-lysine hydroxysuccinimide ester (**26**) was obtained from commercial suppliers.

### Toxicity and transport in *Leishmania donovani*

**Figure 5:**
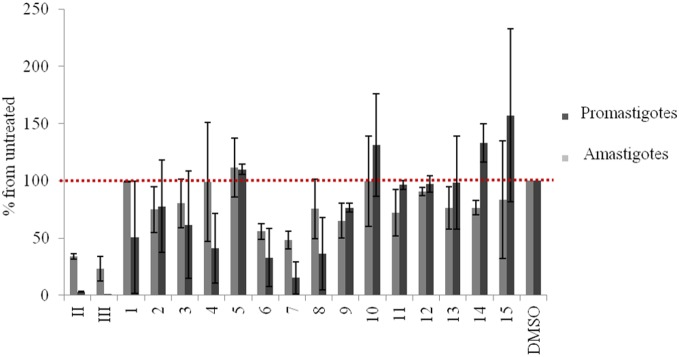
Relative toxicity of conjugates 1–15 and parent compounds II and III in axenic amastigotes and promastigotes. Axenic cultures of *L. donovani* promastigotes and amastigotes were exposed at compound concentration of 1 µg/mL and 2.5 µg/mL, respectively. Thymidine incorporation was measured to monitor cell proliferation. The results show the mean of 2 or 3 independent repeats.

Dose-response analysis was carried out in axenic *L. donovani* promastigotes and amastigotes. To assess the relative toxicity, we first determined the IC_90_ values of the synthesized compounds, as well as those of the parent compounds **II** and **III**. Exposing *L. donovani* to 1 µg/mL **II** or **III** for 48 h killed 95% of promastigotes, whereas a higher concentration (2.5 µg/mL) was required to obtain a similar effect in amastigotes. Therefore, we decided to test the amino acid conjugates **1–15** at the latter concentrations (Fig. 5). All lysine analogues (**1, 6, 11**) were generally less toxic than the parent compounds. Among them, the most effective was **6** that killed 70% of promastigotes and 50% of amastigotes. **1** retained toxicity for promastigotes (50%), but had no effect on amastigotes. **11** was not toxic to promastigotes at all, and was slightly toxic (30%) to amastigotes.

**Figure 6:**
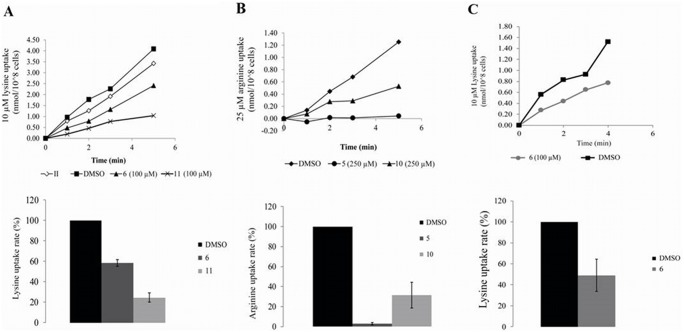
Effect of quinone-amino acid conjugates 5, 6, 10, and 11 on amino acid transport in *L. donovani.* (A) Time course analysis of 10 µM lysine transport in promastigotes in the presence of 100 µM **6** or **11** (Top). Results are also shown in the form of initial rate of transport of two repeats (Bottom); (B) time course analysis of 25 µM arginine transport in promastigotes in the presence of 250 µM **5** or **10** (Top). Initial rate of transport of two repeats (Bottom); (C) time course analysis of 10 µM lysine transport in amastigotes in the presence of 100 µM **6** (Top). Initial rate of transport of two repeats (Bottom). The results show the mean of 2 or 3 independent repeats.

To examine whether the lysine conjugates target lysine transporters we conducted competition experiments to measure transport of lysine in the presence of either **6** or **11**. When treating promastigotes with **6** and **11** (in a ratio 1∶10 lysine:compound) initial transport rates were reduced to 58% and 25% of control (Table 1, Fig. 6A). In amastigotes treated with **6**, initial transport rate was reduced to 47% (Fig. 6C). More importantly, we found that inhibition was dose-dependent (see the 2∶1 ratio column in Table 1). This indicated that the compounds are recognized by the lysine transporter and therefore inhibit lysine transport activity.

**Table 1:** Effect of selected quinone-amino acid conjugates on amino acid transport in *L. donovani* promastigotes.

			Amino acid: Compound ratio			
			(relative transport activity %)*			
Amino acid	Compound	Code	5∶1	2∶1	1∶1	1∶10
**Proline 1 mM**	**2**	2-GABA	32%	-	13%	-
	**7**	7-GABA	49%	-	26%	-
**Lysine 10 µM**	**6**	6-Lys	-	81%	-	58%
	**11**	11-Lys	-	118%	-	25%
**Arginine 25 µM**	**5**	5-Arg	108%	-	33%	3%
	**10**	10-Arg	-	-	-	31%
	**15**	15-Arg	-	-	42%	9%

*Values are shown in percentage, where 100% is the transport rate with no compound added.

Arginine conjugates were even less toxic than lysine derivatives. Indeed, in **5**, **10** and **15** conjugation abolished **II** and **III** toxicity (Fig. 5). However, all three compounds had a strong and dose-dependent effect on arginine transport activity (Table 1, Fig. 6B).

Interestingly, GABA conjugate **7** retained the toxicity of the parent compounds against promastigotes, whereas toxicity against amastigotes was reduced to 50%. On the other hand, **2** or **12** completely lacked the toxicity of the parent compounds (Fig. 5). GABA is a close analogue of proline and has been shown to be transported through the *Leishmania* proline-alanine transporter [46]. To assess whether GABA conjugates **2** and **7** enter promastigotes and amastigotes through LdAAP24, we assayed their inhibitory effect on proline transport. Both **2** and **7** dramatically inhibited proline transport in promastigotes, in spite of the difference in toxicity (Table 1).

**Figure 7:**
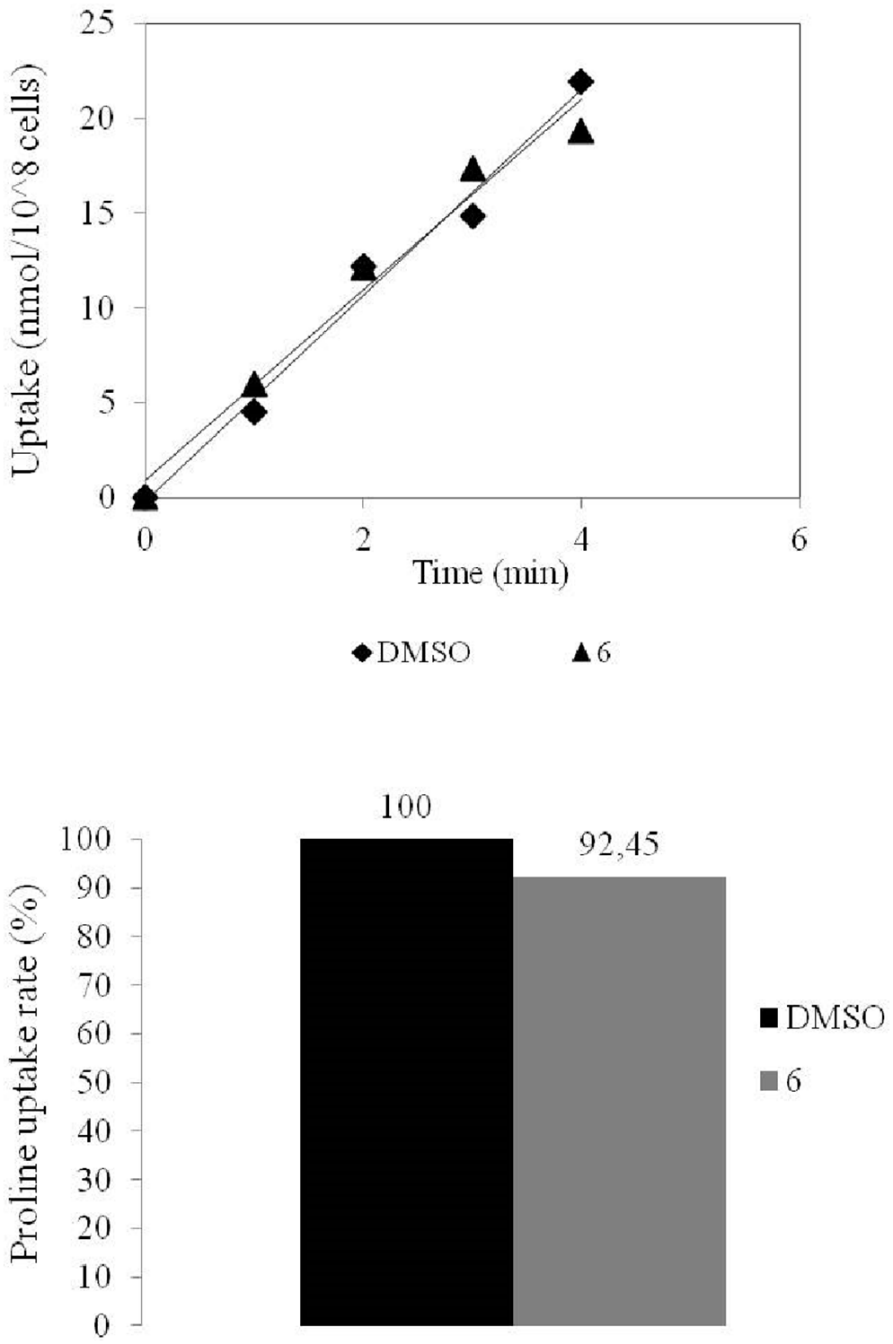
Effect of quinone-amino acid conjugate 6 on proline transport in *L. donovani*. Time course analysis of 1 mM proline in the presence of 100 µM **6** (Top). Results are also shown in the form of initial rate of transport (Bottom), and are the mean of 2 or 3 independent repeats.

In order to validate the hypothesis that transport inhibition was caused by competition between the compounds and the respective amino acids, and not by a general deterioration in cell viability, we also examined the effect of lysine conjugate **6** on the transport of proline. Since lysine is transported by a different transporter than that of proline, there should be no effect of **6** on proline transport. Indeed, we found that proline transport rate was not affected by **6** (Fig. 7), confirming that the general condition of the cells is not compromised by compound toxicity.

Ornithine conjugate **8** showed reasonable toxicity for promastigotes (70%, Fig. 5), but toxicity for amastigotes was reduced to 30%. The ornithine analogues **3** and **13** were significantly less toxic than the parent compounds **II** and **III**.

Since **II** and **III** are hydrophobic, it is likely that they enter parasite cells via simple diffusion. The conjugation with hydrophilic amino acids makes these compounds virtually impermeable by diffusion, thus forcing them to enter cells via membrane transporters.

However, both arginine and lysine transporters are highly specific and sensitive to even minor changes in the substrate structures. Nevertheless, transport results indicated that the compounds successfully compete with the natural substrate of the transporters. This could be the result of blocking the recognition site, and not necessarily the result of entry into the cell. LdAAP24 is less substrate-specific, and we speculated that the conjugates would have been translocated more efficiently by this transporter. Indeed, **7** is the most toxic compound among all the conjugates, and a very effective transport inhibitor. In details, **7** was 5–10 fold more efficient than the tested lysine (**6** and **11**) and arginine (**5**, **10** and **15**) conjugates (Table 1).

**Figure 8:**
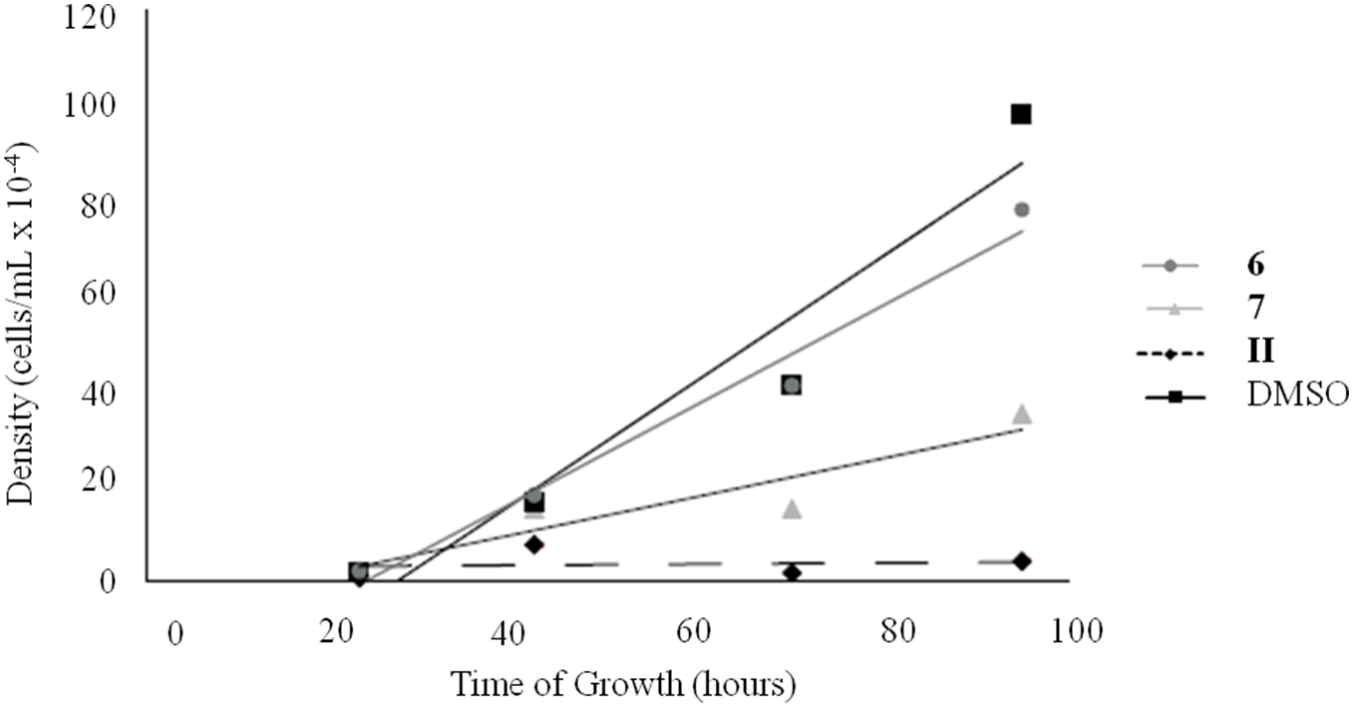
Relative toxicity of 6, 7 and II in THP1 human monocytic cell line. THP1 cells’ growth rate in the presence of **6**, **7** and **II** was compared to that of the control cells grown in the presence of DMSO. THP1 cells were exposed to a compound concentration of 2.5 µg/mL. Cells were counted at several time points until cultures reached stationary phase. Results represent two independent biological repeats, exhibiting the same trend.

To preliminary assess the therapeutic potential of the most toxic compounds against *Leishmania*, citotoxicity of **6** and **7** in THP1 human macrophage cell line, which serves as *ex vivo* model for leishmaniasis, was evaluated. The results depicted in Figure 8 clearly indicated that **7** was still toxic to macrophages, though to a lesser extent compared to the parent molecule **II**. Conversely, **6** did not display any toxicity. These findings nicely suggest that **6**, thanks to its selective cytotoxicity towards *Leishmania* amastigotes with respect to the permissive host cell line, is a potential hit for therapeutic application. Conversely, **7**, which is not able to effectively discriminate among parasite and mammalian cells, is not worthy of further therapeutic pursuit.

## Discussion

Transporters are transmembrane proteins that regulate the translocation of non-diffusible small molecules in and out of cells. Most transporters that are actively being pursued in drug discovery, are localized in the central nervous system, and have been targeted by tricyclic antidepressants since the ‘70s. In the anti-parasitic field, transporters have been much less exploited, even though a few have been showed to translocate Trypanosomatid-toxic compounds [47]. In *Leishmania* amino acid transporters play a major role in creating and maintaining intracellular pools of their corresponding substrates [38]. Hence, conjugating toxic drugs to *Leishmania* nutrients, which exploit specific amino acid transporters, might lead to successful approach for targeted toxicity. However, it should be highlighted that this applies only when the structural features essential for activity are preserved in the conjugates. In our case, it is likely that this might occur, as the parent compounds and the conjugates share the same quinone substructure. Indeed, the quinone core, in addition to a possible target-related mechanism, exerts a toxic effect through a general free-radical-generation mechanism [6].

For many years, the design of transporter-directed chemical probes has led to the identification of specific membrane permeases and to the elucidation of the underlying molecular transport mechanism [48]. In absence of crystallographic or NMR structural data, chemical probing has been used as a major mean for functional identification of targeted transport systems.

Hence, properly designed small molecules might be particularly useful for dissecting the role of the recently identified amino acid transporters in *Leishmania*, both from a drug discovery and a chemical biology viewpoint. To achieve this, we designed a new series of chemical probes able to target *Leishmania* transporters, utilizing an amino acid motif conjugated to a quinone moiety taken from **II** and **III**. The conjugates were evaluated for their toxicity against *Leishmania* and for their ability to modulate arginine, lysine and proline transport activity. Our analysis indicated that the parent compounds **II** and **III** were highly toxic, likely due to their ability to diffuse into cells as hydrophobic compounds. In contrast, all the conjugates were either not toxic or less toxic than **II** and **III**, suggesting that they are no longer permeable to cells due to the hydrophilic amino acid pendants. Notably, our studies experimentally verified that the conjugates interfered with amino acid transport. We hypothesize that the toxic conjugates are translocated by the specific transporters. Once inside the parasite cells, they might accumulate and the quinone fragment can exert its toxic activity. It may appear from our data that the compounds act as antileishmanial agents through substrate competition and not necessarily through the compounds’ toxicity feature. While arginine and lysine are both essential amino acids for the parasites and preventing their entrance would indeed suffice to inhibit parasite growth, proline is not. Therefore, if toxicity was caused only by competition for transport rather than by the toxicity of the compound itself, GABA conjugates inhibiting proline transport should have exhibited lower toxicity as they inhibit the transport of a non-essential amino acid. In light of this, it is likely that the compounds mechanism of action involves activity of the quinone fragment rather than mere competition by the amino acid fragment.

In our view, the fact that the compounds have lower efficacy in amastigotes (mammalian-stage of the parasite) than in promastigotes (insect stage), as well as their decreased toxicity compared to that of **II** and **III**, is compensated for by their improved specificity. In other words, although the compounds are less effective against amastigotes, they are still effective enough to be therapeutically useful. More importantly, they are selectively directed at the parasites’ cells and more toxic to them than to the host, as demonstrated for the lysine conjugate **6**.

This investigation, although preliminary, suggests that *Leishmania* amino acid transporters, in both the extracellular (promastigotes) and intracellular (amastigotes) forms, can serve as drug delivery tools. As far as we know, this is the first time that small molecules modulating such *Leishmania* transporters have been reported; thus, they act as chemical probes useful to study such transporters as drug targets.

In conclusion, the results presented herein may be a starting point for increasing our fundamental understanding of the biological role of *L. donovani* amino acid transporters and of their potential role as molecular targets in anti-trypanosomatid drug discovery. We are confident that further exploration in both these key issues may ultimately contribute to combating leishmaniases with high efficacy and less host toxicity.

## Supporting Information

File S1 **Combined supporting information file containing characterization data for intermediates 16–21, 27–30, 34, 36 and Table S1.** Table S1, Elemental analyses for conjugates **1–15**.
(DOCX)
