# Supplementary material for: Quinone-Amino Acid Conjugates Targeting Leishmania Amino Acid Transporters
Source: PLoS One. 2014 Sep 25;9(9):e107994. doi: 10.1371/journal.pone.0107994 (PMC4177859; doi:10.1371/journal.pone.0107994)
Supplement: File S1 — Combined supporting information file containing characterization data for intermediates 16–21, 27–30, 34, 36 and Table S1. Table S1, Elemental analyses for conjugates 1–15. (DOCX) [file pone.0107994.s001.docx]

**Supporting Information**

**File S1 Combined supporting information file containing characterization data for intermediates 16**-**21**, **27**-**30**, **34**, **36 and Table S1**

Quinone-amino acid conjugates probing *Leishmania* amino acid transporters

**Federica Prati^1,2#^, Adele Goldman-Pinkovich^3#^, Federica Lizzi^1^, Federica Belluti^1^, Roni Koren^3^, Dan Zilberstein^3^, Maria Laura Bolognesi^1^***

**1** Department of Pharmacy and Biotechnology – Alma Mater Studiorum University of Bologna, Bologna, Italy, **2** Department of Drug Discovery and Development, Italian Institute of Technology, Genoa, Italy, **3** Faculty of Biology, Technion-Israel Institute of Technology, Haifa, Israel

^#^These authors contributed equally to the experimental work

*e-mail: [marialaura.bolognesi@unibo.it](mailto:marialaura.bolognesi@unibo.it) tel: +39 0512099717

**Table of Contents**

| Characterization data for intermediates **16**-**21**, **27**-**30**, **34**, and **36** | S2-S4 |
| --- | --- |
| Table S1: elemental analysis for conjugates **1**-**15** | S5 |

2-(4-aminophenylamino)naphthalene-1,4-dione (**16**)

Purple solid; 92% yield; mp: 155-160 °C; ^1^H NMR (CDCl_3_, 200 MHz): δ 3.78 (br s, exchangeable with D_2_O, 2H), 6.22 (s, 1H), 6.74 (d, *J* = 8.4, 2H), 7.09 (d, *J* = 8.4, 2H), 7.47 (br s, exchangeable with D_2_O, 1H), 7.67-7.82 (m, 2H), 8.11 (s, 1H), 8.14 (s, 1H); MS (ESI^+^) *m/z*: 265 (M + H)^+^.

2-(4-(aminomethyl)phenylamino)naphthalene-1,4-dione (**17**)

Red solid; 94% yield; mp: 155-160 °C; ^1^H NMR (CDCl_3_, 400 MHz): δ 1.51 (br s, exchangeable with D_2_O, 2H), 3.89 (s, 2H), 6.39 (s, 1H), 7.25 (d, *J* = 8.4, 2H), 7.38 (d, *J* = 8.4, 2H), 7.55 (br s, exchangeable with D_2_O, 1H), 7.67 (t, *J* = 7.6, 1H), 7.76 (t, *J* = 7.6, 1H), 8.10-8.13 (m, 2H); MS (ESI^+^) *m/z*: 279 (M + H)^+^.

2-(4-(aminomethyl)benzylamino)naphthalene-1,4-dione (**18**)

Orange solid; 92% yield; mp: 162-165 °C. ^1^H NMR (CDCl_3_, 200 MHz): δ 1.66 (br s, exchangeable with D_2_O, 2H), 3.90 (s, 2H), 4.39 (d, *J* = 5.4, 2H), 5.77 (s, 1H), 6.21 (br s, exchangeable with D_2_O, 1H), 7.30 (d, *J* = 8.0, 2H), 7.38 (d, *J* = 8.0, 2H), 7.60-7.83 (m, 2H), 8.05-8.08 (m, 2H); MS (ESI^+^) *m/z*: 293 (M + H)^+^.

*tert*-butyl 4-(1,4-dioxo-1,4-dihydronaphthalen-2-ylamino)phenylcarbamate (**19**)

Black solid; 58% yield. ^1^H NMR (CDCl_3_, 200 MHz): δ 1.50 (s, 9H), 6.29 (s, 1H), 6.81 (br s, exchangeable with D_2_O, 1H), 7.04 (d, *J* = 8.4, 2H), 7.48 (d, *J* = 8.4, 2H), 7.56 (br s, exchangeable with D_2_O, 1H), 7.64-7.83 (m, 2H), 8.05-8.08 (m, 2H); MS (ESI^+^) *m/z*: 365 (M + H)^+^.

*tert*-butyl 4-(1,4-dioxo-1,4-dihydronaphthalen-2-ylamino)benzylcarbamate (**20**)

Red solid; 54% yield. ^1^H NMR (CDCl_3_, 400 MHz): δ 1.47 (s, 9H), 4.32 (s, 2H), 4.88 (br s, exchangeable with D_2_O, 1H), 6.39 (s, 1H), 7.23 (d, *J* = 8.4, 2H), 7.33 (d, *J* = 8.4, 2H), 7.54 (br s, exchangeable with D_2_O, 1H), 7.65-7.69 (m, 1H), 7.74-7.78 (m, 1H), 8.09-8.11 (m, 2H); MS (ESI^+^) *m/z*: 379 (M + H)^+^.

*tert*-butyl 4-((1,4-dioxo-1,4-dihydronaphthalen-2-ylamino)methyl)benzylcarbamate (**21**)

Orange solid; 48% yield. ^1^H NMR (CDCl_3_, 200 MHz): δ 1.48 (s, 9H), 4.33-4.40 (m, 4H), 4.85 (br s, exchangeable with D_2_O, 1H), 5.78 (s, 1H), 6.21 (br s, exchangeable with D_2_O, 1H), 7.27-7.31 (m, 4H), 7.68-7.80 (m, 2H), 8.07-8.13 (m, 2H); MS (ESI^+^) *m/z*: 393 (M + H)^+^.

2,5-dioxopyrrolidin-1-yl 4-(*tert*-butoxycarbonylamino)butanoate (**27**)

White solid; 85% yield; ^1^H NMR (CDCl_3_, 200 MHz): δ 1.46 (s, 9H), 1.90-2.04 (m, 2H), 2.69 (t, *J* = 7.4, 2H), 2.86 (s, 4H), 3.20-3.30 (m, 2H), 4.75 (br s, exchangeable with D_2_O, 1H); MS (ESI^+^) *m/z*: 301 (M + H)^+^.

2,5-dioxopyrrolidin-1-yl-2,2,13,13-tetramethyl-4,11-dioxo-3,12-dioxa-5,10-diazatetradecane-6 carboxylate (**28**)

White solid; 60% yield; ^1^H NMR (CDCl_3_, 400 MHz): δ 1.40 (s, 9H), 1.42 (s, 9H), 1.55-2.01 (m complex, 4H), 2.80 (s, 4H), 3.13-3.15 (m, 2H), 5.12-5.15 (m, 1H); MS (ESI^+^) *m/z*: 430 (M + H)^+^.

2,5-dioxopyrrolidin-1-yl 6-(*tert*-butoxycarbonylamino)hexanoate (**29**)

White solid; 92% yield; ^1^H NMR (CDCl_3_, 200 MHz): δ 1.40 (s, 9H), 1.85 (m, 2H), 1.96 (m, 4H), 2.47 (t, *J* = 6.8, 2H), 2.76 (s, 4H), 2.97 (t, *J* = 7.0, 2H); MS (ESI^+^) *m/z*: 329 (M + H)^+^.

2,5-dioxopyrrolidin-1-yl 6-(*tert*-butoxycarbonylamino)-2,2,15,15-tetramethyl-4,13-dioxo-3,14-dioxa-5,7,12-triazahexadec-5-ene-11-carboxylate (**30**)

White solid; 44% yield; ^1^H NMR (CDCl_3_, 200 MHz): δ 1.43 (s, 9H), 1.46 (s, 9H), 1.49 (s, 9H), 1.58-2.01 (m complex, 4H), 2.80 (s, 4H), 3.92-4.17 (m complex, 2H), 5.96-6.01 (m, 1H); MS (ESI^+^) *m/z*: 572 (M + H)^+^.

2,2,13,13-tetramethyl-4,11-dioxo-3,12-dioxa-5,10-diazatetradecane-6-carboxylic acid (**34**)

White crystals; 85% yield; ^1^H NMR (CDCl_3_, 400 MHz): δ 1.37 (s, 9H), δ 1.45 (s, 9H), 1.56-1.72 (m complex, 4H), 3.13-3.20 (m, 2H), 4.36 (br s, exchangeable with D_2_O, 1H), 4.77 (br s, exchangeable with D_2_O, 1H), 5.21-5.23 (m, 1H); MS (ESI^+^) *m/z*: 333 (M + H)^+^.

6-(*tert*-butoxycarbonylamino)-2,2,15,15-tetramethyl-4,13-dioxo-3,14-dioxa-5,7,12-triazahexadec-5-ene-11-carboxylic acid (**36**)

White crystals; 76% yield; ^1^H NMR (CDCl_3_, 200 MHz): δ 1.46 (s, 9H), 1.51 (s, 9H), 1.54 (s, 9H), 1.71-1.80 (m complex, 4H), 3.80-3.88 (m, 2H), 5.76-5.79 (m, 1H), 9.33 (br s, exchangeable with D_2_O, 1H); MS (ESI^+^) *m/z*: 475 (M + H)^+^.

**Table S1:** Elemental analysis for conjugates **1**-**15**.

| compd | Formula (Mol. Wt.) | Calcd | Found |
| --- | --- | --- | --- |
| 1 | C_22_H_24_N_4_O_3_.2CF_3_COOH | C, 50.33; H, 4.22; N, 9.03 | C, 50.63; H, 4.06; N, 9.21 |
| 2 | C_20_H_19_N_3_O_3_.CF_3_COOH | C, 57.02; H, 4.35; N, 9.07 | C, 57.34; H, 4.62; N, 9.40 |
| 3 | C_21_H_22_N_4_O_3_.2CF_3_COOH.H_2_O | C, 48.08; H, 4.20; N, 8.97 | C, 48.35; H, 4.47; N, 8.65 |
| 4 | C_22_H_23_N_3_O_3_.CF_3_COOH | C, 58.65; H, 4.92; N, 8.55 | C, 58.35; H, 4.72; N, 8.42 |
| 5 | C_22_H_24_N_6_O_3_.2CF_3_COOH.H_2_O | C, 46.85; H, 4.23; N, 12.61 | C, 46.58; H, 4.38; N, 12.87 |
| 6 | C_23_H_26_N_4_O_3_.2CF_3_COOH.H_2_O | C, 49.70; H, 4.63; N, 8.59 | C, 49.60; H, 4.78; N, 8.33 |
| 7 | C_21_H_21_N_3_O_3_.CF_3_COOH | C, 57.86; H, 4.64; N, 8.80; | C, 57.72; H, 4.48; N, 8.65 |
| 8 | C_22_H_24_N_4_O_3_.2CF_3_COOH | C, 50.33; H, 4.22; N, 9.03 | C, 50.65; H, 4.43; N, 9.24 |
| 9 | C_23_H_25_N_3_O_3_.CF_3_COOH | C, 59.40; H, 5.18; N, 8.31 | C, 59.21; H, 5.35; N, 8.52 |
| 10 | C_23_H_26_N_6_O_3_.2CF_3_COOH.H_2_O | C, 47.65; H, 4.44; N, 12.35 | C, 47.82; H, 4.35; N, 12.68 |
| 11 | C_24_H_28_N_4_O_3_.2CF_3_COOH.H_2_O | C, 50.45; H, 4.84; N, 8.41 | C, 50.23; H, 4.49; N, 8.66 |
| 12 | C_22_H_23_N_3_O_3_.CF_3_COOH | C, 58.65; H, 4.92; N, 8.55 | C, 58.37; H, 4.59; N, 8.44 |
| 13 | C_23_H_26_N_4_O_3_.2CF_3_COOH | C, 51.11; H, 4.45; N, 8.83 | C, 51.32; H, 4.68; N, 8.55 |
| 14 | C_24_H_27_N_3_O_3_.CF_3_COOH | C, 60.11; H, 5.43; N, 8.09 | C, 60.34; H, 5.11; N, 8.35 |
| 15 | C_24_H_28_N_6_O_3_.2CF_3_COOH.H_2_O | C, 48.42; H, 4.64; N, 12.10 | C, 48.54; H, 4.31; N, 12.38 |
